# Influence of the PDMS substrate stiffness on the adhesion of *Acanthamoeba castellanii*

**DOI:** 10.3762/bjnano.5.152

**Published:** 2014-08-28

**Authors:** Sören B Gutekunst, Carsten Grabosch, Alexander Kovalev, Stanislav N Gorb, Christine Selhuber-Unkel

**Affiliations:** 1 Institute for Materials Science, Dept. Biocompatible Nanomaterials, Christian-Albrechts-Universität zu Kiel, Kaiserstr. 2, D-24143 Kiel, Germany; 2 Zoological Institute, Dept. Functional Morphology and Biomechanics, Christian-Albrechts-Universität zu Kiel, Am Botanischen Garten 9, D-24118 Kiel, Germany

**Keywords:** acanthamoeba, cell adhesion, elastic substrates, mechanosensing, silicones

## Abstract

**Background:** Mechanosensing of cells, particularly the cellular response to substrates with different elastic properties, has been discovered in recent years, but almost exclusively in mammalian cells. Much less attention has been paid to mechanosensing in other cell systems, such as in eukaryotic human pathogens.

**Results:** We report here on the influence of substrate stiffness on the adhesion of the human pathogen *Acanthamoebae castellanii (A. castellanii)*. By comparing the cell adhesion area of *A. castellanii* trophozoites on polydimethylsiloxane (PDMS) substrates with different Young’s moduli (4 kPa, 29 kPa, and 128 kPa), we find significant differences in cell adhesion area as a function of substrate stiffness. In particular, the cell adhesion area of *A. castellanii* increases with a decreasing Young’s modulus of the substrate.

**Conclusion:** The dependence of *A. castellanii* adhesion on the elastic properties of the substrate is the first study suggesting a mechanosensory effect for a eukaryotic human pathogen. Interestingly, the main targets of *A. castellanii* infections in the human body are the eye and the brain, i.e., very soft environments. Thus, our study provides first hints towards the relevance of mechanical aspects for the pathogenicity of eukaryotic parasites.

## Introduction

The adhesion of many cell types, including fibroblasts, myocytes, and neurons, depends on the mechanical properties of the cellular microenvironment [1–3]. In particular, cells prefer to adhere to materials, which have mechanical properties similar to the ones found in their natural biological environments. Cells can even adapt their direction of migration on materials with gradually changing stiffness, a phenomenon known as mechanotaxis [4–5]. This adaptation is presumably due to an active probing of the cellular microenvironment by nanobiomechanical mechanisms in cells, allowing them to reorient and position themselves [6]. Once grown on a substrate with defined elasticity, cells adapt their own elasticity to the elasticity of their environment [7]. But not only differentiated cells are influenced by substrate stiffness. For stem cells it has been demonstrated that their differentiation is directed towards certain cell types if their adhesion substrate has similar mechanical properties as the natural tissue of the differentiated cell [8], probably mediated by stress-fibre polarization [9]. Even the adhesion of tumor cells is controlled by substrate stiffness [10]. However, not only substrate stiffness plays a decisive role for controlling cell adhesion on soft substrates, but also the specific mechanical anchorage of adhesion molecules [11]. The mechanosensory function of cells is supposed to be closely linked to the mechanisms of active force generation in cells. Analyzing cellular traction forces on elastic substrates has led to substantial information on the forces that cells are able to exert [12–13]. Taken together, there is evidence for the existence of mechanosensors in mammalian cells, yet the detailed mechanisms of mechanosensing are still under investigation. Currently, there are several molecules, such as talin and vinculin as well as ion channels under discussion to serve as possible candidates involved in sensing the mechanical properties of the cellular microenvironment [14–16].

In contrast to mammalian cells, for eukaryotic protists, such as amoebae, there is still only very little knowledge about their ability to sense the elastic properties of their extracellular microenvironments. Only for intracellular mechanosensing, a recent study discusses the signficiant role of myosin-II motor proteins in mechanosensing of the social amoeba *Dictyostelium discoideum* [17]. A medically highly relevant amoeba species are acanthamoebae. *Acanthamoeba spp.* are free-living protists, which are frequently found in water reservoirs such as lakes, swimming-pools, and even in tap water [18]. Some acanthamoeba species are pathogenic to humans, such as *A. culbertsoni* and *A. castellanii* [19–20]. Whereas both *A. culbertsoni* and *A. castellanii* can cause granulomatous amoebic encephalitis (GAE), a chronic and severe disease of the central nervous system [21], *A. castellanii* is more feared for its potential to infect contact lens users and cause a painful and hardly treatable keratitis in their eyes [22]. Such an acanthamoeba keratitis is often related to wrong contact lens care, e.g., due to non-satisfactory contact lens disinfection [23].

In the study presented here we investigated the influence of substrate stiffness on adhesion properties of *A. castellanii.* We prepared polydimethylsiloxane (PDMS) substrates with Young’s moduli of 4 kPa, 29 kPa, and 128 kPa. These Young’s moduli were chosen in order to cover an elasticity range, for which a significant effect of substrate stiffness on the adhesion of mammalian cells has already been reported [1]. We systematically investigated the adhesion of *A. castellanii* on these materials by analyzing the number of adhering amoebae and their adhesion area as a function of substrate stiffness. We demonstrate that the adhesion area of *A. castellanii* is significantly larger on soft substrates compared to stiff substrates, showing the relevance of the cellular microenvironment and associated nanobiomechanical cues also for the adhesion of a eukaryotic human pathogen.

## Experimental

### Preparation of polydimethylsiloxane (PDMS) substrates

Silicone base and curing agent (Sylgard 184, DOW Corning) were mixed thoroughly in a ratio (*m*/*m*) of 80:1, 57:1, and 40:1 by following the curing procedure given in Trappmann et al. [11]. Afterwards, the mixtures were poured in sterile 6-well plates (Sarstedt, Nümbrecht, Germany) up to a thickness of approx. 2 mm and degased for 3.5 h. Thermal polymerization was carried out for 21 h at 70 °C followed by a slow cool down to room temperature.

### Elasticity measurements

Mechanical properties of PDMS substrates were determined by microindentation using a micro-force measurements device (Basalt-BT01, Tetra GmbH, Ilmenau, Germany) [24]. The recorded force–distance curves were used to calculate the Young’s modulus of the PDMS substrates and the work of adhesion with the Johnson–Kendall–Roberts (JKR) model [25]. This model is used to characterize the mechanical properties of soft materials in the presence of adhesion, since it takes into account the attractive forces between the microindenter tip and the sample. For all substrates, elastic moduli were determined from the unloading part of the curve to consider only the elastic behavior and not the plastic deformation of the sample. The measurements were performed under ambient conditions (25–26 °C temperature and 40–50% relative humidity). Analysis of the Young’s moduli of the different PDMS substrates resulted in 4 ± 1 kPa (silicone base/curing agent = 80:1), 29 ± 3 kPa (57:1), and 128 ± 32 kPa (40:1).

### Acanthamoeba culture

Trophozoites of *A. castellanii* (ATTC 30234) were cultured at room temperature in Peptone Yeast Glucose (PYG) 712 medium (20.0 g proteose peptone (BD, Sparks, USA), 1.00 g yeast extract (BD, Sparks, USA), 950 mL distilled water, 10.0 mL 0.4 M MgSO_4_·7H_2_O (AppliChem, Darmstadt, Germany), 8.00 mL 0.05 M CaCl_2_ (AppliChem, Darmstadt, Germany), 34.0 mL 0.1 M sodium citrate dihydrate (Merck, Darmstadt, Germany), 10.0 mL 0.005 M (NH_4_)_2_Fe(SO_4_)_2_·6H_2_O (AppliChem, Darmstadt, Germany), 10.0 mL 0.25 M Na_2_HPO_4_·7H_2_O (Roth, Karlsruhe, Germany), 10.0 mL 0.25 M KH_2_PO_4_ (Roth, Karlsruhe, Germany), 50.0 mL 2 M glucose (Sigma–Aldrich Chemie GmbH, Steinheim, Germany)). In this axenic culture, the PYG 712 medium was regularly exchanged in the cell culture flasks in order to avoid an encystment of *A. castellanii* trophozoites.

### Adhesion experiments

The PDMS substrates were washed with PYG 712 medium before use. *A. castellanii* were detached from the cell culture substrate by cautiously hitting the culture flask. The acanthamoebae were counted by a Neubauer hemocytometer and 30.000 acanthamoebae were incubated in 1 mL PYG 712 for 1 h to ensure that the amoebae are fully spread at the time of the experiment. After this incubation period, 30 phase-contrast images were captured with a 10× objective (UPlanFL, Olympus, Japan) on an inverted microscope (IX-81, Olympus, Japan) for each PDMS substrate and for the control substrate (sterile 6-well plate, Sarstedt, Nümbrecht, Germany) by using a digital camera (C-9300, Hamamatsu, Japan). The experiments were carried out on three different days (on each day in triplicate). Cell numbers and areas were evaluated by manual image segmentation with ImageJ [26]. Statistical significance was analyzed by using a Kruskal–Wallis test and a multiple comparison test in Matlab (MathWorks, USA).

## Results and Discussion

**Figure 1:**
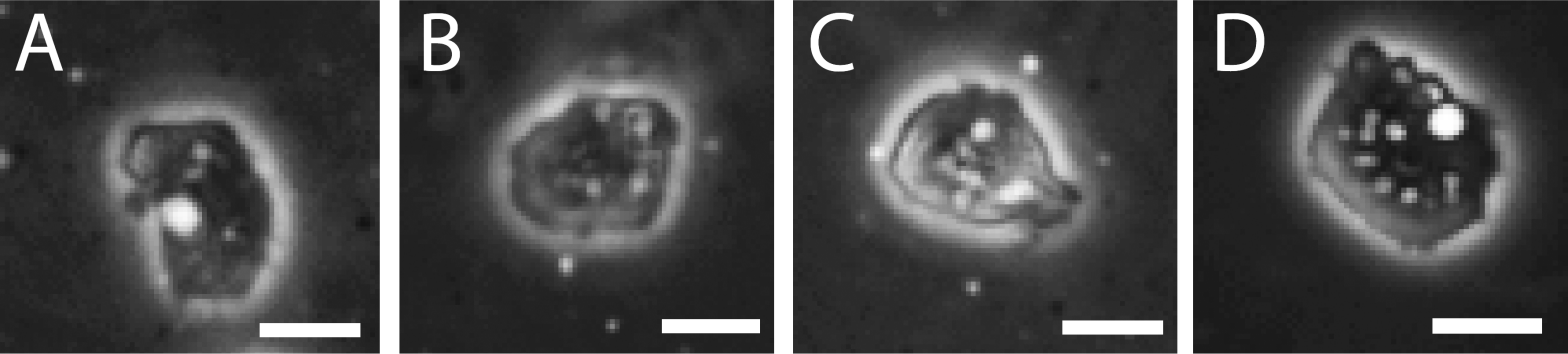
Phase contrast images of *A. castellanii* trophozoites on PDMS substrates with different Young’s moduli and a control after 1 h in culture in PYG medium: (A) 4 kPa, (B) 29 kPa, (C) 128 kPa, and (D) control. The adhesion area of *A. castellanii* is influenced by substrate stiffness, i.e., the cell area on the stiff sample (C) is smaller than on the softer samples (A,B) and on the control sample (D). The comparison of acanthamoeba morphology on PDMS substrates and on the control sample shows that acanthamoebae adhere very well to the non-functionalized PDMS substrates. Scale bar: 15.2 µm.

Polydimethylsiloxane (PDMS) has, in recent years, proven to be a versatile tool for cell adhesion studies, in particular for studying effects of microstructures on cell adhesion [27–28], and it is also well-known for its excellent biocompatibility [29–30]. Figure 1 shows typical phase-contrast images of *A. castellanii* trophozoites adhering to the PDMS substrates and to the control substrate used in this study. The phase contrast images reveal strong halos that surround the acanthamoebae. This means that the acanthamoebae do not flatten during spreading on neither of the substrates, but keep an ellipsoidal shape. Thus, they do not spread as extensively as many mammalian cell types [31–32]. Images of *A. castellanii* on substrates with different Young’s moduli as well as on a tissue culture control sample (Figure 1) show that the adhesion of *A. castellanii* is, at first glance, not strongly influenced by the substrate stiffness. But a closer look reveals differences: On substrates with a low Young’s modulus (4 kPa), *A. castellanii* occupy a larger area compared to acanthamoebae on substrates with a higher Young’s modulus (128 kPa). The substrate with a Young’s modulus of 29 kPa gave an intermediate value. On the control sample, the cell adhesion area was similar to the one on the 4 kPa substrate. The increase of adhesion area with decreasing Young`s modulus is opposite to the behavior of human mesenchymal stem cells [33] but is in good agreement with the trend observed in studies on neural stem cell cultures [34]. This result is reasonable, as during the infection process, *A. castellanii* adhere to comparably soft microenvironments in the brain and in the eye. Interestingly, there is no significant difference in the morphology of *A. castellanii* between PDMS substrates and the positive control substrate. This shows that *A. castellanii* trophozoites can adhere very well to PDMS without the need for further biofunctionalization, as the PDMS was used in its hydrophobic, non-functionalized state. This is very important to note, as recently, indications have been found that not only the elasticity of the substrate is a decisive parameter for cell adhesion, but instead also the linkage of adhesion molecules to the substrate [11]. Due to the usage of non-functionalized PDMS substrates, we can therefore exclude such an effect of adhesion-ligand anchorage in our experiments.

**Figure 2:**
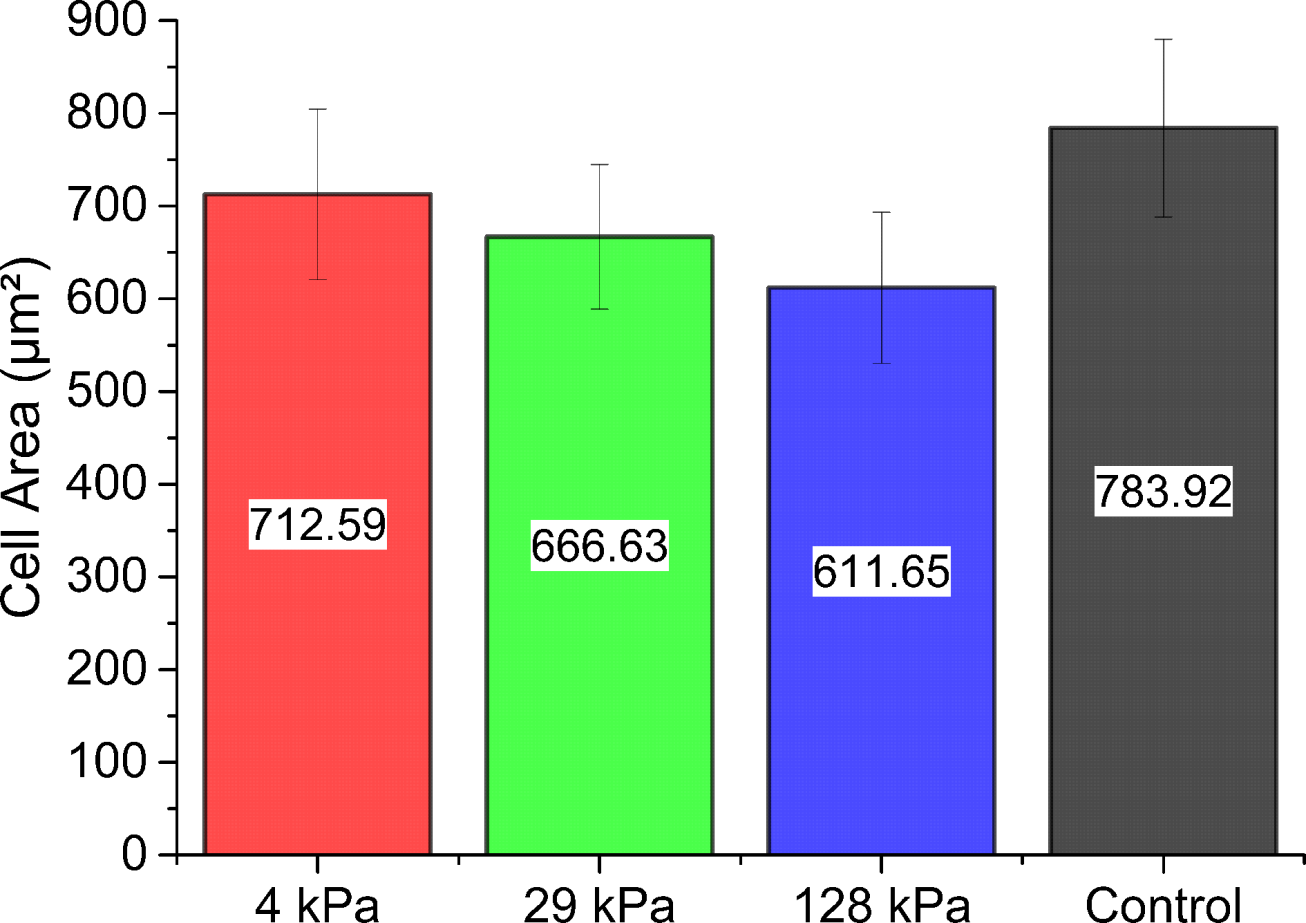
Cell adhesion area of *A. castellanii* as a function of the Young’s modulus of the PDMS substrates and in comparison to the control substrate after 1 h of adhesion in PYG medium. These results were obtained from analyzing 3092 amoebae (4 kPa), 3044 amoebae (29 kPa), 3108 amoebae (128 kPa), and 2194 amoebae (control). The bar diagram gives the mean cell area (calculated from the mean of cell adhesion area on each substrate) and standard deviation. This standard deviation is a measure for the differences in cell adhesion area on different individual samples of the same type. The numeric mean values are additionally given inside the bars. The differences of the means are statistically significant (Kruskal–Wallis test; ***, *p* < 0.001, *n* > 2194 cells per substrate type).

**Figure 3:**
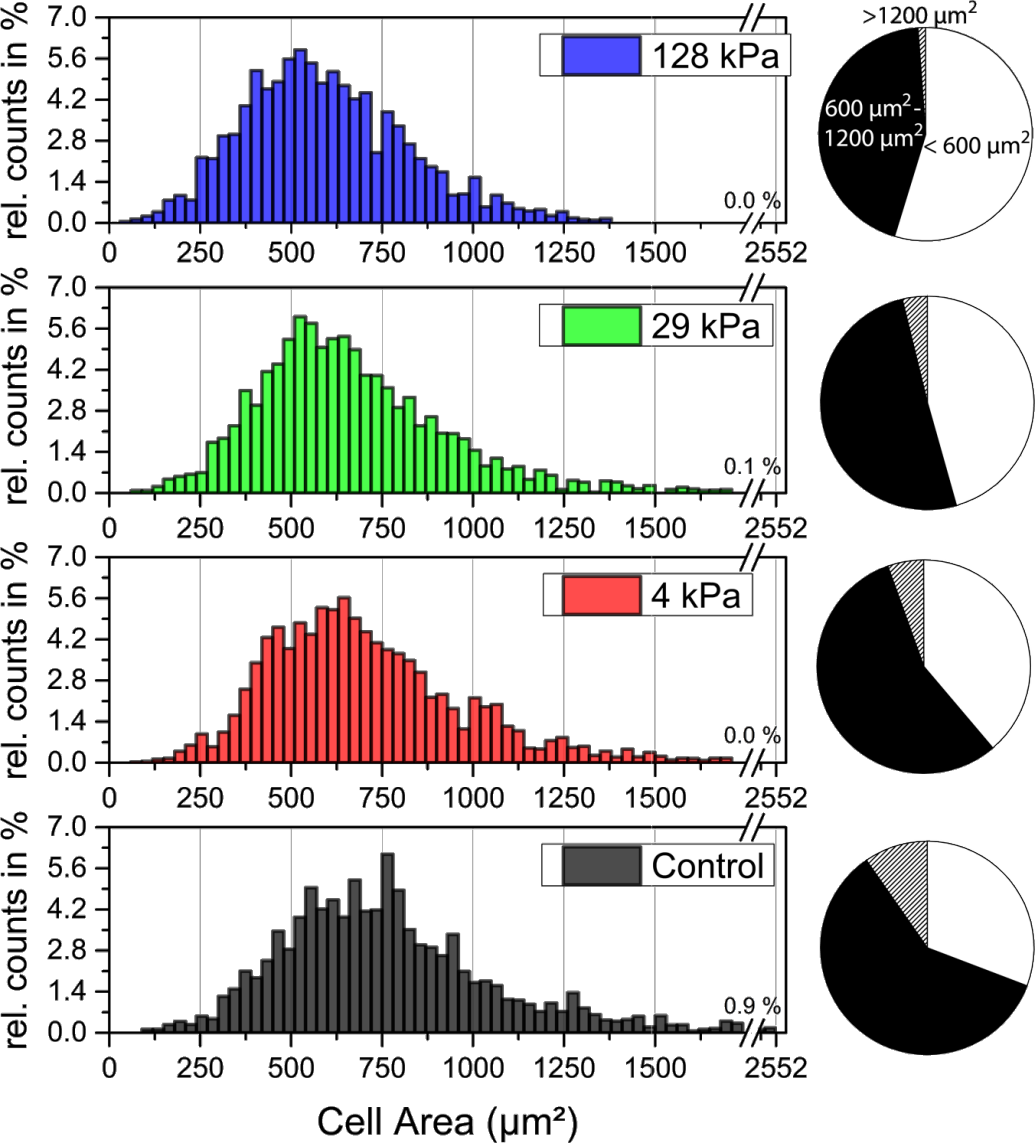
Average relative counts of projected cell areas of adhering *A. castellanii* on PDMS substrates and on the control substrates. The histograms show that the distribution is slightly asymmetric and can therefore not be fitted with a Gaussian. Average relative counts were calculated by determining the relative counts per sample and generating the average for each bin from all experiments, in order to equally rate all experiments. The value above the interception of the x-axis shows the relative counts of cell adhesion areas larger than 1750 µm^2^. Differences in cell area distribution become particularly visible when comparing the pie charts (white: cell adhesion area < 600 µm^2^; black: 600 µm^2^ ≤ cell adhesion area ≤ 1200 µm^2^; striped: cell adhesion area > 1200 µm^2^).

In order to characterize the dependence of the cell area on the substrate stiffness in further detail, we carried out an extensive analysis of the cell areas of *A. castellanii* by image segmentation of several thousands of acanthamoebae per substrate type. The results of this analysis are summarized in Figure 2 and Figure 3. Figure 2 shows the mean values and standard deviations for the projected cell adhesion areas of *A. castellanii* as a function of the substrate stiffness. These values were determined from nine experiments in total, i.e., three independent experiments carried out on three independent measurement days. The data clearly show that the cell adhesion area decreases with increasing substrate stiffness of PDMS substrates. Statistical analysis revealed that all mean ranks are significantly different at a 0.001 level (***).

In Figure 3, we present the distribution of projected cell areas as a function of substrate stiffness compared to the control sample. Interestingly, the distribution of projected cell areas does not follow a Gaussian distribution function, but shows a tail towards large cell areas. This tail of the distribution is a very typical feature of cell sizes, and has been reported for many cell types, such as mammalian cells [35–36], but also for *A. castellanii* [37]. Comparing the distribution of cell adhesion areas for substrates having different Young’s moduli supports the results from Figure 1 and Figure 2, i.e., that substrate stiffness influences cell adhesion area. The difference between the control sample and the PDMS sample with Young’s modulus 128 kPa is eye-catching here, but differences can also be observed between the different PDMS samples. In particular the pie charts demonstrate that the amount of large and small cells changes as a function of PDMS stiffness.

**Figure 4:**
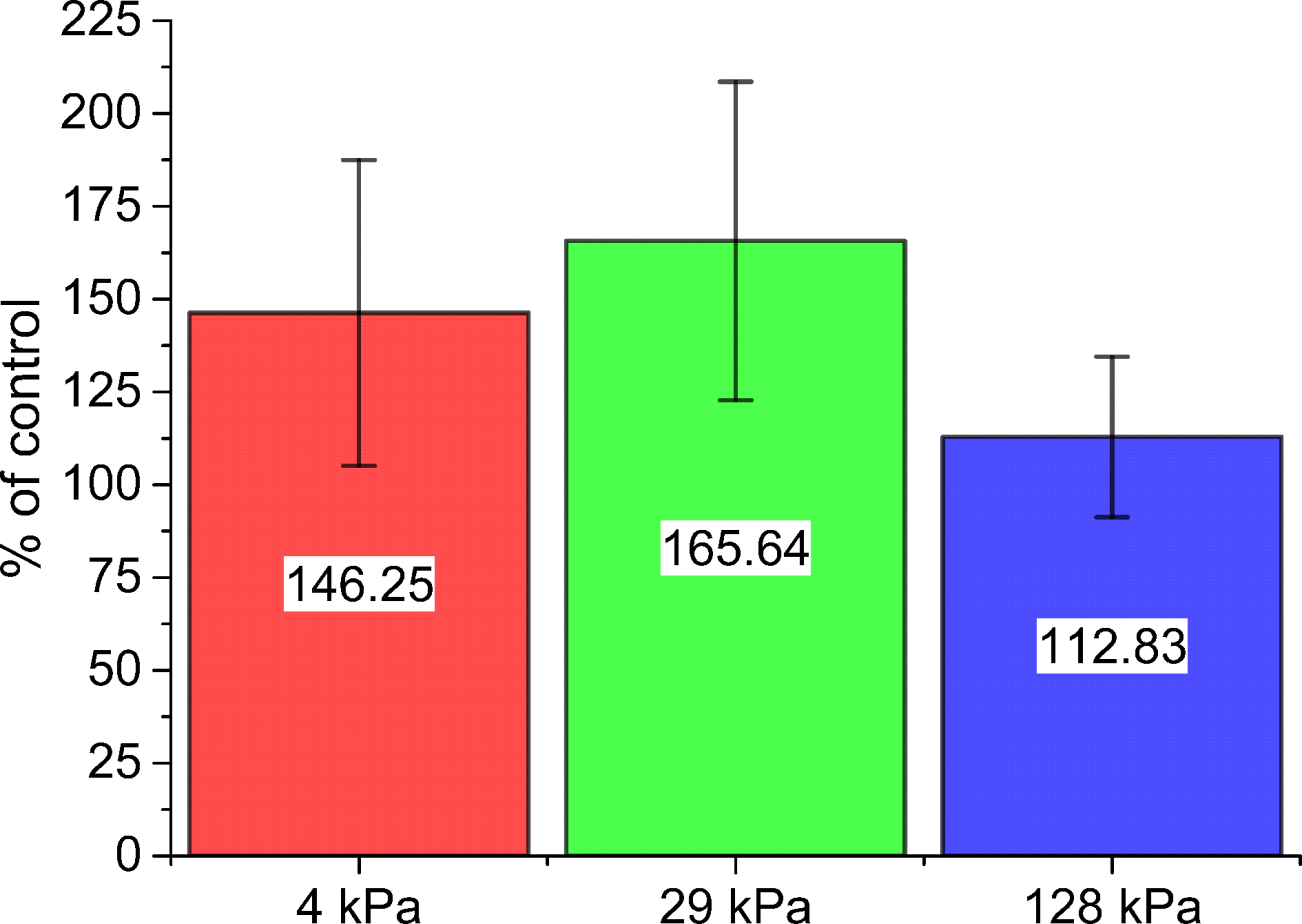
Numbers (in % of control) of *A. castellanii* adhering to PDMS substrates after 1 h of incubation. The values were normalized to the number of *A. castellanii* adhering to the control substrate. Here, no systematic effect of the substrate stiffness on the number of cells could be observed. Mean values are shown in a bar diagram, where each numeric value is given inside the bar. Error bars denote standard deviations.

The dependence of the adhesion on the substrate stiffness is, however, not reflected in the number of acanthamoebae attached to the different surfaces (Figure 4). No systematic relation for the dependence of the number of attached acanthamoebae on the substrate stiffness could be found in our experiments after 1 h of adhesion. The incubation time of 1 h chosen here might be too short to generate severe impact on parameters such as cell proliferation, as the doubling time of acanthamoeba in axenic culture is on the timescale of days [38]. As acanthamoeba can also be grown in suspension [19], their proliferation might not be strongly influenced by the presence of any substrate.

In a recent study, we had investigated the adhesion of *A. castellanii* to hydrogel materials used in contact lenses [39]. We determined a very strong dependence of *A. castellanii* adhesion on the water content of contact lens materials, i.e., a strong increase in adhesion with increasing water content. In the study presented here, the PDMS material was hydrophobic and repelled water. Also according to literature, the water content of PDMS is negligible [40]. Therefore, a bias of our data by changes in the water content of the substrates can be excluded for the experiments presented here. Furthermore, in our previous study, we did not find a significant dependence of the adhesion on the substrate stiffness for Young’s moduli between 0.30 and 0.66 MPa [39]. In contrast to this previous study, we here discuss adhesion dependence on Young’s moduli that are one order of magnitude smaller. However, it seems logical that *A. castellanii* spread larger on soft substrates, as their main human infection targets, eye and brain, are very soft, with the brain having Young’s moduli of about 1–10 kPa and less [41]. However, the influence of the substrate stiffness on the adhesion of *A. castellanii* that we observe in our study is not as pronounced as for mammalian cells [2]. Such an extenuated effect is reasonable, as acanthamoebae have to be able to survive and migrate in very diverse natural environments, ranging from soil to water reservoirs.

## Conclusion

We have presented an adhesion analysis of human pathogenic *A. castellanii* to soft elastic substrates. We find that the cell adhesion areas of *A. castellanii* change significantly as a function of the substrate stiffness, with the largest average cell adhesion areas present on the softest substrates with a Young’s modulus of 4 kPa. In contrast, the number of adhering acanthamoebae is not significantly changed by the substrate stiffness after 1 h of adhesion. Our results indicate that adhesion of *A. castellanii* is influenced by substrate stiffness, presumably by mechanosensory mechanisms that allow them to sense and react on the stiffness of their surrounding environment. Our study provides first evidence for such a mechanosensory function in the adhesion of *A. castellanii.* Furthermore, mammalian cells are known to adhere preferably to substrates with mechanical properties similar to their natural environment. We show that *A. castellanii* adhere with larger contact areas on softer substrates. This is very interesting, as their natural targets in the human body are soft environments (brain, eye). Therefore, our study also shows very first indications for the relevance of mechanical aspects in the pathogenicity of parasites and can serve as a starting point for many future studies on the impact of mechanical parameters on the adhesion of pathogenic organisms.
